# Supplementary material for: Cholinergic input to mouse visual cortex signals a movement state and acutely enhances layer 5 responsiveness
Source: eLife. 2024 Jul 26;12:RP89986. doi: 10.7554/eLife.89986 (PMC11281783; doi:10.7554/eLife.89986)
Supplement: Supplementary file 1. — For statistical comparisons we used paired or unpaired t-tests, or hierarchical bootstraps as indicated in the table below. The units (ROIs, sites, or mice) over which testing was done are boldfaced. For hierarchical bootstraps the two levels of units used are boldfaced. [file elife-89986-supp1.docx]

Supplementary File 1

| Figure panel | Value compared | Mean 1 | SD 1 | Mean 2 | SD 2 | Test | P-value | N (ROIs) | N (Sites) | N (Mice) |
| --- | --- | --- | --- | --- | --- | --- | --- | --- | --- | --- |
| 1K | Fraction of responsive cholinergic axons responsive to locomotion onsets *vs* grating onsets | 0.523 | 0.220 | 0.063 | 0.075 | t-test | <10^-5^ | - | **25** | 14 |
|  | Fraction of responsive cholinergic axons responsive to locomotion onsets *vs* mismatch onsets | 0.523 | 0.220 | 0.062 | 0.051 | t-test | <10^-5^ | - | **25** | 14 |
|  | Fraction of responsive cholinergic axons responsive to locomotion onsets | 0.523 | 0.220 | - | - | t-test | <10^-5^ | - | **25** | 14 |
|  | Fraction of responsive cholinergic axons responsive to grating onsets | 0.063 | 0.075 | - | - | t-test | 0.40 | - | **25** | 14 |
|  | Fraction of responsive cholinergic axons responsive to mismatch onsets | 0.062 | 0.051 | - | - | t-test | 0.25 | - | **25** | 14 |
| 2A | Locomotion velocity at locomotion bout onset *vs* offset | 0.020 | 0.008 | 0.014 | 0.005 | Hierarchical bootstrap | 0 | **5048** | **25** | 14 |
| 2B | Cholinergic axon activity [% ΔF/F] at locomotion bout onset *vs* offset | 1.47 | 2.03 | 1.39 | 1.80 | Hierarchical bootstrap | 0.22 | **5048** | **25** | 14 |
| 2C | Correlation between locomotion bout onset and offset for cholinergic axon activity *vs* locomotion velocity | 0.554 | 0.215 | 0.135 | 0.258 | t-test | <10^-5^ | - | **25** | 14 |
| 2E | Average cholinergic axon activity [% ΔF/F] for low *vs* intermediate locomotion velocity | 1.01 | 0.022 | 1.01 | 0.022 | Hierarchical bootstrap | 0.58 | **5048,3657** | **25,**  **17** | 14,  11 |
|  | Average cholinergic axon activity [% ΔF/F] for intermediate *vs* high locomotion velocity | 1.01 | 0.022 | 1.01 | 0.008 | Hierarchical bootstrap | 0.59 | **3657,**  **1282** | **17,**  **7** | 11,  5 |
|  | Average cholinergic axon activity [% ΔF/F] for low *vs* high locomotion velocity | 1.01 | 0.022 | 1.01 | 0.008 | Hierarchical bootstrap | 0.70 | **5048,**  **1282** | **25,**  **7** | 14,  5 |
| 2F | Correlation of activity of cholinergic axons with raw *vs* binarized velocity | 0.083 | 0.082 | 0.099 | 0.092 | Hierarchical bootstrap | 0 | **5048** | **25** | 14 |
| 3A | Correlation of average cholinergic axon activity with locomotion velocity in closed loop *vs* open loop | 0.286 | 0.170 | 0.322 | 0.199 | t-test | 0.15 | - | **25** | 14 |
|  | Correlation of average cholinergic axon activity with locomotion velocity in dark *vs* grating | 0.319 | 0.200 | 0.311 | 0.167 | t-test | 0.77 | - | **25** | 14 |
|  | Correlation of average cholinergic axon activity with locomotion velocity in closed loop *vs* dark | 0.286 | 0.170 | 0.319 | 0.200 | t-test | 0.27 | - | **25** | 14 |
|  | Correlation of average cholinergic axon activity with locomotion velocity in open loop *vs* grating | 0.322 | 0.199 | 0.311 | 0.167 | t-test | 0.75 | - | **25** | 14 |
|  | Correlation of average cholinergic axon activity with locomotion velocity in open loop *vs* dark | 0.322 | 0.199 | 0.319 | 0.200 | t-test | 0.93 | - | **25** | 14 |
|  | Correlation of average cholinergic axon activity with locomotion velocity in closed loop *vs* grating | 0.286 | 0.170 | 0.311 | 0.167 | t-test | 0.33 | - | **25** | 14 |
| 3B | Correlation of average cholinergic axon activity with pupil diameter in closed loop *vs* open loop | 0.165 | 0.150 | 0.277 | 0.170 | t-test | 0.0001 | - | **25** | 14 |
|  | Correlation of average cholinergic axon activity with pupil diameter in dark *vs* grating | 0.002 | 0.141 | 0.300 | 0.136 | Unpaired  t-test | 0.0000 | - | **21,**  **25** | 13,  14 |
|  | Correlation of average cholinergic axon activity with pupil diameter in closed loop *vs* dark | 0.165 | 0.150 | 0.002 | 0.141 | Unpaired  t-test | 0.0005 | - | **25,**  **21** | 14,  13 |
|  | Correlation of average cholinergic axon activity with pupil diameter in open loop *vs* grating | 0.277 | 0.170 | 0.300 | 0.136 | t-test | 0.61 | - | **25** | 14 |
|  | Correlation of average cholinergic axon activity with pupil diameter in open loop *vs* dark | 0.277 | 0.170 | 0.002 | 0.141 | Unpaired  t-test | 0.0000 | - | **25,**  **21** | 14,  13 |
|  | Correlation of average cholinergic axon activity with pupil diameter in closed loop *vs* grating | 0.165 | 0.150 | 0.300 | 0.136 | t-test | 0.0028 | - | **25** | 14 |
| 3C | Correlation of pupil diameter with locomotion velocity in closed loop *vs* open loop | 0.324 | 0.217 | 0.457 | 0.215 | t-test | 0.0001 | - | **25** | 14 |
|  | Correlation of pupil diameter with locomotion velocity in dark *vs* grating | 0.060 | 0.232 | 0.435 | 0.239 | Unpaired  t-test | 0.0000 | - | **21,**  **25** | 13,  14 |
|  | Correlation of pupil diameter with locomotion velocity in closed loop *vs* dark | 0.324 | 0.217 | 0.060 | 0.232 | Unpaired  t-test | 0.0003 | - | **25,**  **21** | 14,  13 |
|  | Correlation of pupil diameter with locomotion velocity in open loop *vs* grating | 0.457 | 0.215 | 0.435 | 0.239 | t-test | 0.70 | - | **25** | 14 |
|  | Correlation of pupil diameter with locomotion velocity in open loop *vs* dark | 0.457 | 0.215 | 0.060 | 0.232 | Unpaired  t-test | 0.0000 | - | **25,**  **21** | 14,  13 |
|  | Correlation of pupil diameter with locomotion velocity in closed loop *vs* grating | 0.324 | 0.217 | 0.435 | 0.239 | t-test | 0.045 | - | **25** | 14 |
| 4A | Grating response difference in  layer 2/3 neurons [% ΔF/F] while locomoting *vs* not locomoting | -0.12 | 10.66 | -1.97 | 6.78 | Hierarchical bootstrap | 0 | **6529** | **21** | 14 |
| 4B | Grating response difference in  layer 5 neurons [% ΔF/F] while locomoting *vs* not locomoting | 3.13 | 8.10 | 0.88 | 5.50 | Hierarchical bootstrap | 0 | **6798** | **25** | 14 |
| 4C | Grating response difference in  layer 2/3 neurons [% ΔF/F] on optogenetic stimulation *vs* no stimulation while not locomoting | -2.43 | 8.10 | -2.44 | 7.44 | Hierarchical bootstrap | 0.46 | **3992** | **13** | 13 |
| 4D | Grating response difference in  layer 5 neurons [% ΔF/F] on optogenetic stimulation *vs* no stimulation while not locomoting | 1.21 | 6.05 | 0.62 | 6.06 | Hierarchical bootstrap | 0.0060 | **4715** | **16** | 13 |
| 4E | Mismatch response difference in layer 2/3 neurons [% ΔF/F] on optogenetic stimulation vs no stimulation | 2.00 | 10.14 | 2.25 | 8.36 | Hierarchical bootstrap | 0.66 | **2506** | **9** | 9 |
| 4F | Mismatch response difference in  layer 5 neurons [% ΔF/F] on optogenetic stimulation *vs* no stimulation | 2.72 | 7.26 | 1.77 | 5.30 | Hierarchical bootstrap | 0.028 | **2801** | **9** | 6 |
| 4G | Locomotion onset response difference in closed loop in layer 2/3 neurons [% ΔF/F] on optogenetic stimulation *vs* no stimulation | 1.66 | 9.36 | 1.23 | 5.76 | Hierarchical bootstrap | 0.13 | **2747** | **10** | 10 |
| 4H | Locomotion onset response difference in closed loop in layer 5 neurons [% ΔF/F] on optogenetic stimulation *vs* no stimulation | 2.63 | 5.54 | 1.86 | 4.26 | Hierarchical bootstrap | 0 | **3623** | **11** | 8 |
| 5A | Mean response in layer 2/3 neurons [% ΔF/F] on locomotion onset *vs* zero | 1.82 | 7.37 | - | - | Hierarchical bootstrap | 0 | **6154** | **20** | 14 |
| 5B | Mean response in layer 5 neurons [% ΔF/F] on locomotion onset *vs* zero | 2.00 | 4.28 | - | - | Hierarchical bootstrap | 0 | **6032** | **22** | 12 |
| 5C | Mean response in layer 2/3 neurons [% ΔF/F] to optogenetic stimulation of ChAT axons *vs* zero | -0.07 | 6.63 | - | - | Hierarchical bootstrap | 0.62 | **6154** | **20** | 14 |
| 5D | Mean response in layer 5 neurons [% ΔF/F] on optogenetic stimulation of ChAT axons *vs* zero | -0.16 | 3.21 | - | - | Hierarchical bootstrap | 0.86 | **6032** | **22** | 13 |
| 6E | Difference in average pairwise correlation of layer 2/3 neurons while locomoting *vs* not locomoting | 0.035 | 0.020 | 0.038 | 0.021 | Hierarchical bootstrap | 0.045 | **7826** | **25** | 15 |
|  | Difference in average pairwise correlation of layer 2/3 neurons on optogenetic stimulation *vs* no stimulation while not locomoting | 0.021 | 0.018 | 0.029 | 0.022 | Hierarchical bootstrap | 0 | **1688** | **4** | 4 |
| 6F | Difference in average pairwise correlation of layer 5 neurons while locomoting *vs* not locomoting | 0.030 | 0.017 | 0.036 | 0.017 | Hierarchical bootstrap | <10^-3^ | **7132** | **26** | 14 |
|  | Difference in average pairwise correlation of layer 5 neurons on optogenetic stimulation *vs* no stimulation while not locomoting | 0.009 | 0.010 | 0.014 | 0.009 | Hierarchical bootstrap | 0.0017 | **100** | **4** | 4 |
| 6G | Layer 2/3 neuron difference in the change in average pairwise correlation on locomotion following ligand injection for DREADD activation *vs* inhibition | -0.0005 | <10^-3^ | -0.002 | <10^-3^ | Hierarchical bootstrap | 0.32 | **2429,**  **3103** | **7,**  **10** | 7,  9 |
| 6H | Layer 5 neuron difference in the change in average pairwise correlation on locomotion following ligand injection for DREADD activation *vs* inhibition | -0.003 | <10^-3^ | 0.007 | <10^-3^ | Hierarchical bootstrap | 0.032 | **1651,**  **1762** | **7,**  **7** | 7,  7 |
| 7A | Latency to grating response onset in layer 2/3 neurons [ms] while locomoting *vs* not locomoting | 596 | 470 | 632 | 451 | Hierarchical bootstrap | 0.86 | **1481** | **24** | 15 |
|  | Latency to grating response onset in layer 2/3 neurons [ms] on optogenetic stimulation *vs* no stimulation while not locomoting | 656 | 427 | 659 | 444 | Hierarchical bootstrap | 0.54 | **1503** | **15** | 15 |
| 7B | Latency to grating response onset in layer 5 neurons [ms] while locomoting *vs* not locomoting | 658 | 413 | 771 | 417 | Hierarchical bootstrap | 0.0037 | **938** | **25** | 14 |
|  | Latency to grating response onset in layer 5 neurons [ms] on optogenetic stimulation *vs* no stimulation while not locomoting | 709 | 407 | 793 | 413 | Hierarchical bootstrap | 0.0046 | **783** | **16** | 13 |
| 1–s1D | Latency to response on locomotion onset [ms] for cholinergic axons *vs* layer 2/3 neurons | 477 | 457 | 366 | 614 | Hierarchical bootstrap | 0.040 | **1182,**  **1166** | **23, 25** | 12, 15 |
|  | Latency to response on locomotion onset [ms] for cholinergic axons *vs* layer 5 neurons | 477 | 457 | 635 | 503 | Hierarchical bootstrap | 0.014 | **1182,**  **1299** | **23,**  **26** | 12,  14 |
|  | Latency to response on locomotion onset [ms] for layer 2/3 neurons *vs* layer 5 neurons | 366 | 614 | 635 | 503 | Hierarchical bootstrap | 0 | **1166,**  **1299** | **25,**  **26** | 15,  14 |
| 2–s1G | Correlation of cholinergic axon population activity vector between bout onset and during the bout for actual *vs* random triggers | 0.735 | 0.187 | 0.007 | 0.088 | t-test | <10^-5^ | - | **25** | 14 |
|  | Correlation of cholinergic axon population activity vector between during the bout and at bout onset for actual *vs* random triggers | 0.759 | 0.194 | 0.036 | 0.120 | t-test | <10^-5^ | - | **25** | 14 |
|  | Correlation of cholinergic axon population activity vector between bout onset and offset for actual *vs* random triggers | 0.721 | 0.196 | 0.012 | 0.103 | t-test | <10^-5^ | - | **25** | 14 |
| 2–s1H | Average GRAB-ACh activity [% ΔF/F] in visual cortex during low *vs* high velocity | 1.3 | 3.4 | 0.22 | 3.9 | Hierarchical bootstrap | 0.7 | **178,**  **132** | **8,6** | 8,6 |
| 3–s1A | Variability in pupil diameter [SD] in closed loop *vs* open loop | 21.32 | 8.47 | 18.68 | 5.48 | t-test | 0.15 | - | **25** | 14 |
|  | Variability in pupil diameter [SD] in dark *vs* grating | 25.52 | 17.45 | 16.54 | 7.16 | Unpaired  t-test | 0.023 | - | **21,**  **25** | 13,  14 |
|  | Variability in pupil diameter [SD] in closed loop *vs* dark | 21.32 | 8.47 | 25.52 | 17.45 | Unpaired  t-test | 0.29 | - | **25,**  **21** | 14,  13 |
|  | Variability in pupil diameter [SD] in open loop *vs* grating | 18.68 | 5.48 | 16.54 | 7.16 | t-test | 0.10 | - | **25** | 14 |
|  | Variability in pupil diameter [SD] in open loop *vs* dark | 18.68 | 5.48 | 25.52 | 17.45 | Unpaired  t-test | 0.070 | - | **25,**  **21** | 14,  13 |
|  | Variability in pupil diameter [SD] in closed loop *vs* grating | 21.32 | 8.47 | 16.54 | 7.16 | t-test | 0.041 | - | **25** | 14 |
| 3–s1B | Correlation of average cholinergic axon activity with locomotion velocity *vs* facial movement in closed loop | 0.286 | 0.170 | 0.095 | 0.172 | t-test | <10^-4^ | - | **25** | 14 |
|  | Correlation of average cholinergic axon activity with locomotion velocity *vs* facial movement in open loop | 0.322 | 0.199 | 0.148 | 0.195 | t-test | 0.0001 | - | **25** | 14 |
|  | Correlation of average cholinergic axon activity with locomotion velocity *vs* facial movement in dark | 0.319 | 0.200 | 0.090 | 0.224 | t-test | <10^-4^ | - | **25** | 14 |
|  | Correlation of average cholinergic axon activity with locomotion velocity *vs* facial movement in grating condition | 0.311 | 0.167 | 0.130 | 0.214 | t-test | 0.0001 | - | **25** | 14 |
| 4–s1A | Mice locomotion velocity [cm/s] on optogenetic stimulation of cholinergic axons, before *vs* after | 2.53 | 3.09 | 2.4 | 3.09 | Hierarchical bootstrap | 0.81 | - | **51,**  **51** | 15,  15 |
| 4–s1B | Average locomotion velocity [cm/s], before *vs* upon optogenetic stimulation of cholinergic axons in visual cortex | 2.53 | 3.09 | 2.4 | 3.09 | Hierarchical bootstrap | 0.81 | - | **51,**  **51** | 15,  15 |
| 4–s2A | Grating response difference in 10% highest grating responsive layer 2/3 neurons [% ΔF/F] on optogenetic stimulation *vs* no stimulation while not locomoting | 4.87 | 12.36 | 5.47 | 11.87 | Hierarchical bootstrap | 0.74 | **399** | **13** | 13 |
| 4–s2B | Mismatch response difference in layer 2/3 neurons [% ΔF/F] on optogenetic stimulation vs no stimulation | 8.47 | 19.46 | 9.57 | 25.84 | Hierarchical bootstrap | 0.68 | **251** | **9** | 9 |
| 4–s2C | Locomotion onset response difference in closed loop in layer 2/3 neurons [% ΔF/F] on optogenetic stimulation *vs* no stimulation | 11.05 | 16.99 | 9.94 | 10.56 | Hierarchical bootstrap | 0.23 | **275** | **10** | 10 |
| 4–s3F | Change in average pairwise correlation of layer 2/3 neuron on control stimulation *vs* no stimulation while not locomoting | 0.025 | 0.030 | 0.025 | 0.022 | Hierarchical bootstrap | 0.46 | **1047** | **3** | 3 |
|  | Change in average pairwise correlation of layer 5 neurons on control stimulation *vs* no stimulation while not locomoting | 0.017 | 0.021 | 0.012 | 0.006 | Hierarchical bootstrap | 0.18 | **88** | **3** | 3 |
| 4–s4A | Mean response in layer 2/3 neurons [% ΔF/F] to grating onset (preferred + π/4 Ori) stationary *vs* locomotion | -3.10 | 9.36 | -0.30 | 9.95 | Hierarchical bootstrap | 0 | **2396, 2500** | **13, 13** | 13, 13 |
|  | Mean response in layer 2/3 neurons [% ΔF/F] to grating onset (preferred Ori) stationary *vs* locomotion | -1.50 | 12.87 | 1.80 | 18.57 | Hierarchical bootstrap | 0 | **3908, 3992** | **13, 13** | 13, 13 |
|  | Mean response in layer 2/3 neurons [% ΔF/F] to grating onset (preferred - π/4 Ori) stationary *vs* locomotion | -2.67 | 9.84 | -0.84 | 11.83 | Hierarchical bootstrap | 0.002 | **2729, 2729** | **13, 13** | 13, 13 |
|  | Mean response in layer 2/3 neurons [% ΔF/F] to grating onset (preferred + π/2 Ori) stationary *vs* locomotion | -2.64 | 9.04 | -0.92 | 13.97 | Hierarchical bootstrap | 0.009 | **2519, 2572** | **13, 13** | 13, 13 |
|  | Mean response in layer 2/3 neurons [% ΔF/F] to grating onset (preferred + π/4 Ori) stationary *vs* (stationary + opto) | -3.10 | 9.36 | -2.50 | 6.95 | Hierarchical bootstrap | 0.08 | **2396, 2500** | **13, 13** | 13, 13 |
|  | Mean response in layer 2/3 neurons [% ΔF/F] to grating onset (preferred Ori) stationary *vs* (stationary + opto) | -1.50 | 12.87 | -1.33 | 10.54 | Hierarchical bootstrap | 0.27 | **3908, 3992** | **13, 13** | 13, 13 |
|  | Mean response in layer 2/3 neurons [% ΔF/F] to grating onset (preferred - π/4 Ori) stationary *vs* (stationary + opto) | -2.67 | 9.84 | -2.59 | 7.61 | Hierarchical bootstrap | 0.42 | **2729, 2729** | **13, 13** | 13, 13 |
|  | Mean response in layer 2/3 neurons [% ΔF/F] to grating onset (preferred + π/2 Ori) stationary *vs* (stationary + opto) | -2.64 | 9.04 | -2.22 | 8.33 | Hierarchical bootstrap | 0.32 | **2519, 2572** | **13, 13** | 13, 13 |
| 4–s4B | Mean response in layer 5 neurons [% ΔF/F] to grating onset (preferred + π/4 Ori) stationary *vs* locomotion | -0.26 | 10.40 | 2.01 | 10.72 | Hierarchical bootstrap | 10^-4^ | **3448,**  **3520** | **16, 16** | 13,  13 |
|  | Mean response in layer 5 neurons [% ΔF/F] to grating onset (preferred Ori) stationary *vs* locomotion | 0.28 | 11.76 | 4.49 | 15.16 | Hierarchical bootstrap | 0 | **4670,**  **4715** | **16, 16** | 13, 13 |
|  | Mean response in layer 5 neurons [% ΔF/F] to grating onset (preferred - π/4 Ori) stationary *vs* locomotion | -0.28 | 9.58 | 2.77 | 9.74 | Hierarchical bootstrap | 0 | **3518,**  **3614** | **16, 16** | 13, 13 |
|  | Mean response in layer 5 neurons [% ΔF/F] to grating onset (preferred + π/2 Ori) stationary *vs* locomotion | -0.83 | 8.85 | 2.33 | 9.66 | Hierarchical bootstrap | 0 | **3642,**  **3642** | **16, 16** | 13, 13 |
|  | Mean response in layer 5 neurons [% ΔF/F] to grating onset (preferred + π/4 Ori) stationary *vs* (stationary + opto) | -0.26 | 10.40 | -0.10 | 7.18 | Hierarchical bootstrap | 0.30 | **3448, 3520** | **16, 16** | 13, |
|  | Mean response in layer 5 neurons [% ΔF/F] to grating onset (preferred Ori) stationary *vs* (stationary + opto) | 0.28 | 11.76 | 1.37 | 9.62 | Hierarchical bootstrap | 0 | **4670, 4715** | **16, 16** | 13,  13 |
|  | Mean response in layer 5 neurons [% ΔF/F] to grating onset (preferred - π/4 Ori) stationary *vs* (stationary + opto) | -0.28 | 9.58 | 0.14 | 7.23 | Hierarchical bootstrap | 0.13 | **3518, 3614** | **16, 16** | 13, 13 |
|  | Mean response in layer 5 neurons [% ΔF/F] to grating onset (preferred + π/2 Ori) stationary *vs* (stationary + opto) | -0.83 | 8.85 | -0.53 | 6.02 | Hierarchical bootstrap | 0.22 | **3642, 3642** | **16, 16** | 13,  13 |
| 6–s2B | Locomotion velocity before *vs* after DREADD activation | 0.022 | 0.005 | 0.028 | 0.005 | t-test | <10^-3^ | - | **14** | 7 |
|  | Locomotion velocity before *vs* after DREADD inhibition | 0.017 | 0.004 | 0.016 | 0.005 | t-test | 0.46 | - | **17** | 9 |
| 6–s2D | Time spent locomoting before *vs* after DREADD activation | 0.683 | 0.143 | 0.699 | 0.154 | t-test | 0.64 | - | **14** | 7 |
|  | Time spent locomoting before *vs* after DREADD inhibition | 0.572 | 0.156 | 0.360 | 0.164 | t-test | <10^-5^ | - | **17** | 9 |
| 6–s2E | Total distance run before *vs* after DREADD activation | 5.086 | 1.964 | 6.511 | 2.457 | t-test | 0.0067 | - | **14** | 7 |
|  | Total distance run before *vs* after DREADD inhibition | 3.314 | 1.713 | 2.144 | 1.413 | t-test | <10^-4^ | - | **17** | 9 |
|  | Change in total distance run on DREADD activation *vs* inhibition | 1.425 | 1.657 | -1.170 | 0.913 | Unpaired  t-test | <10^-5^ | - | **14, 17** | 7,  9 |
| 6–s3A | Change in average pairwise correlation on locomotion in layer 2/3 neurons in DREADD activation group pre-CNO *vs* zero | -0.010 | 0.019 | - | - | Hierarchical bootstrap | 0 | **2429** | **7** | **7** |
|  | Change in average pairwise correlation on locomotion in layer 2/3 neurons in DREADD activation group post-CNO *vs* zero | -0.010 | 0.019 | - | - | Hierarchical bootstrap | 10^-4^ | **2429** | **7** | **7** |
|  | Change in average pairwise correlation on locomotion in layer 2/3 neurons in DREADD inhibition group pre-CNO *vs* zero | -0.004 | 0.019 | - | - | Hierarchical bootstrap | 0.01 | **3103** | **10** | 9 |
|  | Change in average pairwise correlation on locomotion in layer 2/3 neurons in DREADD inhibition group post-CNO *vs* zero | -0.006 | 0.020 | - | - | Hierarchical bootstrap | 0.02 | **3103** | **10** | 9 |
| 6–s3B | Change in average pairwise correlation on locomotion in layer 5 neurons in DREADD activation group pre-CNO *vs* zero | -0.008 | 0.016 | - | - | Hierarchical bootstrap | 0 | **1651** | **7** | **7** |
|  | Change in average pairwise correlation on locomotion in layer 5 neurons in DREADD activation group post-CNO *vs* zero | -0.011 | 0.019 | - | - | Hierarchical bootstrap | 0.003 | **1651** | **7** | **7** |
|  | Change in average pairwise correlation on locomotion in layer 5 neurons in DREADD inhibition group pre-CNO *vs* zero | -0.010 | 0.020 | - | - | Hierarchical bootstrap | 0.001 | **1762** | **7** | **7** |
|  | Change in average pairwise correlation on locomotion in layer 5 neurons in DREADD inhibition group post-CNO *vs* zero | -0.002 | 0.016 | - | - | Hierarchical bootstrap | 0.23 | **1762** | **7** | **7** |
