# Supplementary material for: Cholinergic input to mouse visual cortex signals a movement state and acutely enhances layer 5 responsiveness
Source: eLife. 2024 Jul 26;12:RP89986. doi: 10.7554/eLife.89986 (PMC11281783; doi:10.7554/eLife.89986)
Supplement: Supplementary file 2. [file elife-89986-supp2.docx]

Supplementary file 2

| Genotype | Virus | Site of injection | Nbr. Mice | Nbr. ROIs | Figures |  |
| --- | --- | --- | --- | --- | --- | --- |
| ChAT-IRES-Cre | | AAV2/5-hSyn1-FLEx-axon-GCaMP6s | Basal forebrain – MS/vDB/hDB | 12 | 4451 | Figures 1, 2, 3, Figure 1–figure supplement 1, Figure 2–figure supplement 1G, Figure 2–figure supplement 2, Figure 3–figure supplement 1 |
| ChAT-IRES-Cre | | AAV PHP.eB-Ef1α-DIO-GCaMP6s | Basal forebrain – MS/vDB/hDB | 2 | 597 | Figures 1, 2, 3, Figure 1–figure supplement 1, Figure 2–figure supplement 1G, Figure 2–figure supplement 2, Figure 3–figure supplement 1 |
| ChAT-IRES-Cre | | AAV2/1-hSyn-DIO-ChrimsonR-tdTomato | Basal forebrain – MS/vDB/hDB | 15 | 15 103 | Figures 4, 5, 6A-6F, 7, Figure 1–figure supplement 1, Figure 4–figure supplement 1, Figure 4–figure supplement 2, Figure 4–figure supplement 3D-F, Figure 4–figure supplement 4, Figure 4–figure supplement 5, Figure 6–figure supplement 1, Figure 7–figure supplement 1 |
|  |  | AAV2/1-Ef1α-GCaMP6f-WPRE | Primary visual cortex |  |  |  |
| ChAT-IRES-Cre | | AAV2/1-Ef1α-DIO-hM3D(Gq)-mCherry | Basal forebrain – MS/vDB/hDB | 7 | 4080 | Figures 6G-6H, Figure 6–figure supplement 2, Figure 6–figure supplement 3 |
|  |  | AAV2/1-Ef1α-GCaMP6f-WPRE | Primary visual cortex |  |  |  |
| ChAT-IRES-Cre | | AAV2/1-Ef1α-DIO-hM4D(Gi)-mCherry | Basal forebrain – MS/vDB/hDB | 9 | 4865 | Figures 6G-6H, Figure 6–figure supplement 2, Figure 6–figure supplement 3 |
|  |  | AAV2/1-Ef1α-GCaMP6f-WPRE | Primary visual cortex |  |  |  |
| ChAT-IRES-Cre | | AAV2/9-hSyn-GRAB-ACh3.0 | Primary visual cortex | 8 | 609 | Figure 2–figure supplement 1A-C and 1H |
| ChAT-IRES-Cre | | AAV2/1-Ef1α-DIO-tdTomato-WPRE | Basal forebrain – MS/vDB/hDB | 5 | 2409 | Figure 4–figure supplement 3A-C |
|  |  | AAV2/1-Ef1α-GCaMP6f-WPRE | Primary visual cortex |  |  |  |
| ChAT-IRES-Cre | | AAV2/1-Ef1α-eGFP-WPRE | Primary visual cortex | 6 | 2640 | Figure 2–figure supplement 1D-F |
